# Differentiation of adipose-derived stem cells into Schwann cell-like cells through intermittent induction: potential advantage of cellular transient memory function

**DOI:** 10.1186/s13287-018-0884-3

**Published:** 2018-05-11

**Authors:** Xun Sun, Yun Zhu, He-yong Yin, Zhi-yuan Guo, Feng Xu, Bo Xiao, Wen-li Jiang, Wei-min Guo, Hao-ye Meng, Shi-bi Lu, Yu Wang, Jiang Peng

**Affiliations:** 1 Institute of Orthopedics, Chinese PLA General Hospital; Beijing Key Lab of Regenerative Medicine in Orthopedics; Key Lab of Musculoskeletal Trauma & War Injuries, PLA, No.28 Fuxing Road, Beijing, 100853 People’s Republic of China; 2 School of Medicine, Nankai University, No.94 Weijin Road, Tianjin, 300071 People’s Republic of China; 3 School of Biomedical Sciences, Li Ka Shing Faculty of Medicine, University of Hong Kong, No.21 Sassoon Road, Pokfulam, 999077 Hong Kong; 4 Department of Surgery, Experimental Surgery and Regenerative Medicine, Ludwig-Maximilians-University (LMU), Nussbaumstr. 20, 80336 Munich, Germany; 5 Department of Ultrasound, Beijing Hospital, National Center of Gerontology, No.1 Dahua Road, Beijing, 100730 People’s Republic of China; 6 Co-innovation Center of Neuroregeneration, Nantong University, Nantong, Jiangsu Province, 226007 People’s Republic of China

**Keywords:** Adipose-derived stem cells, Schwann cell-like cells, Peripheral nerve regeneration, Cell transplantation, Differentiation, Intermittent induction

## Abstract

**Background:**

Peripheral nerve injury (PNI) is a worldwide issue associated with severe social and economic burden. Autologous nerve grafting, the gold standard treatment for peripheral nerve defects, still has a number of technical limitations. Tissue engineering technology is a novel therapeutic strategy, and mesenchymal stromal cells (MSCs) are promising seed cells for nerve tissue engineering. However, the efficiency of traditional methods for inducing the differentiation of MSCs to Schwann cell-like cells (SCLCs) remains unsatisfactory.

**Methods:**

Here, we propose an intermittent induction method with alternate use of complete and incomplete induction medium to induce differentiation of adipose-derived stem cells (ASCs) to SCLCs. The time dependence of traditional induction methods and the efficiency of the intermittent induction method and traditional induction methods were evaluated and compared using immunocytochemistry, quantitative reverse transcription polymerase chain reaction (qRT-PCR), enzyme-linked immunosorbent assay (ELISA), and co-culture with the dorsal root ganglion (DRG) in vitro. Cell transplantation was used to compare the effects of the traditional induction method and the intermittent induction method in repairing sciatic nerve defects in vivo.

**Results:**

The results of the present study indicated that the intermittent induction method is more efficient than traditional methods for inducing ASCs to differentiate into SCLCs. In addition, SCLCs induced by this method were closer to mature myelinating Schwann cells and were capable of secreting neurotrophins and promoting DRG axon regeneration in vitro. Furthermore, SCLCs induced by the intermittent induction method could repair sciatic nerve defects in rats by cell transplantation in vivo more effectively than those produced by traditional methods.

**Conclusion:**

Intermittent induction represents a novel strategy for obtaining seed cells for use in nerve tissue engineering.

**Electronic supplementary material:**

The online version of this article (10.1186/s13287-018-0884-3) contains supplementary material, which is available to authorized users.

## Background

Peripheral nerve injury (PNI) affects 1 million people every year worldwide [1, 2]. There are nearly 300,000 traumatic PNI cases in Europe every year. In addition, PNI accounts for as many as 3% of all trauma patients in the United States, which increases to 5% if plexus and root avulsions are taken into consideration [3, 4]. End-to-end suturing is sufficient for treatment in cases of simple nerve transection. However, in the majority of cases, simple suturing is not applicable because of the large distance between proximal and distal stumps. Autologous nerve grafting is the gold standard treatment for this type of PNI [5, 6]. Autologous nerves provide a physical and biological scaffold to induce axon growth through the defect area, thus reconstructing the continuity of nerves [7, 8]. There are a number of technical limitations in autologous nerve grafting, such as limited donor sources, complications from second operations, and risk of painful neuroma formation [5, 9, 10]. Therefore, peripheral nerve repair using synthetic or bio-derived transplants has aroused widespread attention.

Tissue engineering technology is a novel therapeutic strategy for peripheral nerve defects [11]. The typical composition of tissue-engineered nerve grafts includes scaffold material, seed cells, and neurotrophic factors. Schwann cells (SCs) or cells with similar functions are promising as seed cells for tissue-engineered nerve grafts. Unfortunately, in clinical application, SCs face difficulties with regard to isolation, proliferation, and purification, as well as donor site complications [12–14]. Moreover, residual fibroblasts remain during SC culture and proliferate much more rapidly than SCs [11]. This would become a severe problem for peripheral nerve regeneration because scars will form if SCs are transplanted along with large numbers of fibroblasts [15]. Therefore, seed cell sources with extensive self-renewal capacity, broad differentiation potential, low immunogenicity, and readily accessible properties are urgently needed [16].

Mesenchymal stromal cells (MSCs), which are derived from self-renewing multipotent adult precursors in the mesoderm germ layer, are promising seed cells for tissue engineering [17]. They have the capacity to differentiate into mesodermal lineage cells, including osteoblasts, chondroblasts, and adipocytes [18]. Indeed, MSCs are heterogeneous and contain several populations, including stem cells. After their adherent culture and cell expansion in vitro, the purified stem cells from MSCs are divided into different categories depending on where they come from, such as bone marrow-derived stem cells (BMSCs) and adipose-derived stem cells (ASCs) [19]. Although BMSCs are the most studied type of MSCs for use in SC differentiation, their clinical applicability is still limited because of their low yield and high degree of invasiveness [20]. The proliferation capability and differentiation potential of BMSCs also decreases with increasing donor age [16]. It has been reported that Schwann cell-like cells (SCLCs) derived from BMSCs and ASCs can promote nerve regeneration. However, ASCs have a number of advantages in comparison with BMSCs, including lower invasiveness of acquisition, abundance, rapid rate of expansion in vitro, and are capable of long-term survival and integration in host tissues with immunological tolerance [21].

ASCs have been shown to be able to differentiate into SCLCs with expression of neurogenic growth factors in vitro which promote myelination and axonal regeneration in PNI animal models [22–24]. The most common means of inducing differentiation of ASCs into SCLCs are the Dezawa and Kingham methods [25, 26]. However, neither of these methods has satisfactory efficiency since the surface markers of SCs, such as S100 and glial fibrillary acidic protein (GFAP), have relatively low expression rates (approximately 30–60% positive). In addition, the apoptosis of many unsuccessfully differentiated ASCs would further reduce S100 and GFAP expression [27–30]. The time span of induction during the sustaining induction period following pre-induction is not consistent in both the Dezawa and Kingham methods [28, 31–33]. Studies have shown that the efficiency of inducing differentiation of BMSCs into SCLCs exhibited time dependence in the sustaining induction period, and the optimal sustaining induction period is 5–6 days [34, 35].

Here, we simulated the process of MSC induction into adipocytes with the application of a novel method to induce differentiation of ASCs into SCLCs by the alternate use of complete and incomplete induction medium. This intermittent induction method can strongly promote differentiation of ASCs into SCLCs, thus providing high-quality seed cells for tissue-engineered nerve grafts.

## Methods

### Animals

The experimental procedures involving laboratory animals were approved by the Institutional Animal Care and Use Committee of the Chinese PLA General Hospital and were performed in accordance with the Guidelines for the Care and Use of Laboratory Animals from the Chinese Ministry of Public Health and US National Institutes of Health. Sprague–Dawley (SD) rats used in the experiments were obtained from the Laboratory Animal Research Center of Chinese PLA General Hospital and housed in a temperature-controlled environment under a 14 h/10 h light/dark cycle. All procedures described below were carried out during the light period of the cycle and all surgical procedures were performed under general anesthesia with intraperitoneal injection of pentobarbital.

### Isolation and culture of adipose-derived stem cells and Schwann cells

ASCs were isolated from the inguinal fat region of 3-week-old SD rats. The fat was then cut into chyliform and digested with collagenase II (1.5 mg/ml; C6885; Sigma) dissolved in Dulbecco’s modified Eagle’s medium/Ham’s F-12 50/50 mix (DMEM/F-12; Corning) for 30 min at 37 °C. The cells were harvested by enzyme digestion and cultured in α-modified minimal essential medium (α-MEM) (SH30265; Hyclone) with 10% fetal bovine serum (FBS) (35–011-CV; Corning) at 37 °C under an atmosphere of 5% CO_2_ following centrifugation and resuspension. ASCs were able to grow adhering to the wall and were further passaged when they had reached 80% confluency. Cells at passage 2 were used for all subsequent experiments.

SCs were harvested from the sciatic nerves of 3-day-old SD rats. Briefly, lengths of approximately 1 cm of the sciatic nerves were cut, and the epineurium was removed under a microscope. The sciatic nerves were then cut into small pieces approximately 1 mm in length and enzymatically dissociated in collagenase NB4 (2 mg/ml; 17,454; SERVA) dissolved in DMEM/F-12 for 15 min at 37 °C, centrifuged, and resuspended in DMEM/F-12 containing 10% FBS. SCs obtained by the above method were cultured in an incubator at 37 °C under an atmosphere of 5% CO_2_. After 48 h, the supernatant was removed and SCs were resuspended in 1 mg/ml collagenase NB4 to purify these cells from fibroblasts. The purified SCs were used for all subsequent experiments.

### Multipotential differentiation of adipose-derived stem cells

ASCs at passage 2 were induced to undergo adipogenic, osteogenic, and chondrogenic differentiation to identify the characteristics of multidifferentiation. For adipogenic differentiation, ASCs were seeded in six-well plates at a density of 2 × 10^5^ cells/well. SD rat adipose-derived stem cell adipogenic differentiation medium (RASMD-90031; Cyagen Biosciences Inc.) was used to induce adipogenic differentiation after cells had reached confluence. The induction/maintenance media were replaced alternately for three cycles of 3 days/1 day. Differentiated cells were fixed and stained with Oil Red O working solution (3:2 dilution with distilled water and Filter with filter paper) for 30 min. For osteogenic differentiation, ASCs were seeded in six-well plates at a density of 1 × 10^5^ cells/well. SD rat adipose-derived stem cell osteogenic differentiation medium (RASMD-90021; Cyagen Biosciences Inc.) was used to induce osteogenic differentiation when cells had reached 70% confluence, and completely replaced by fresh medium every 3 days for 3 weeks. Differentiated cells were fixed and stained with Alizarin Red working solution for 5 min. For chondrogenic differentiation, ASCs were resuspended in medium following digestion with 0.25% trypsin (25,200,056; Gibco), and aliquots of 5 × 10^5^ cells were centrifuged in 15-ml polypropylene culture tubes. SD rat adipose-derived stem cell chondrogenic differentiation medium (RASMD-90042; Cyagen Biosciences Inc.) was added to the cell pellet used to induce chondrogenic differentiation at 37 °C in a humidified atmosphere of 5% CO_2_ after discarding the supernatant. The complete chondrogenic medium was replaced every 3 days for 21 days. Chondrogenic pellets were fixed in 4% paraformaldehyde (P1110; Solarbio) and 5-μm thick paraffin sections were cut. The sections were stained with Toluidine Blue O solution (1% in sodium borate; G3663; Solarbio) for 5 min. Images were captured using a microscope equipped with a DP71 camera (BX51; Olympus).

### Flow cytometry

ASCs were resuspended following digestion with 0.25% trypsin and washed three times with phosphate-buffered saline (PBS). A minimum of 1 × 10^5^ cells were collected from each sample. Samples were incubated with phycoerythrin (PE)-coupled antibodies against rat CD29 (562,154; BD Biosciences), CD34 (sc-7324; Santa Cruz), CD45 (554,878; BD Biosciences), CD86 (551,396; BD Biosciences), and CD90 (551,401; BD Biosciences), and fluorescein isothiocyanate (FITC)-coupled antibody against rat CD44 (550,974; BD Biosciences) in the dark at room temperature for 30 min. Nonspecific isotype-matched antibodies were used as controls. The samples were subjected to flow cytometry, and data were analyzed using Paint-A-Gate Pro™ software (FACSCalibur™; BD Biosciences).

### Experimental design and differentiation into Schwann cell-like cells

**Fig. 1:**
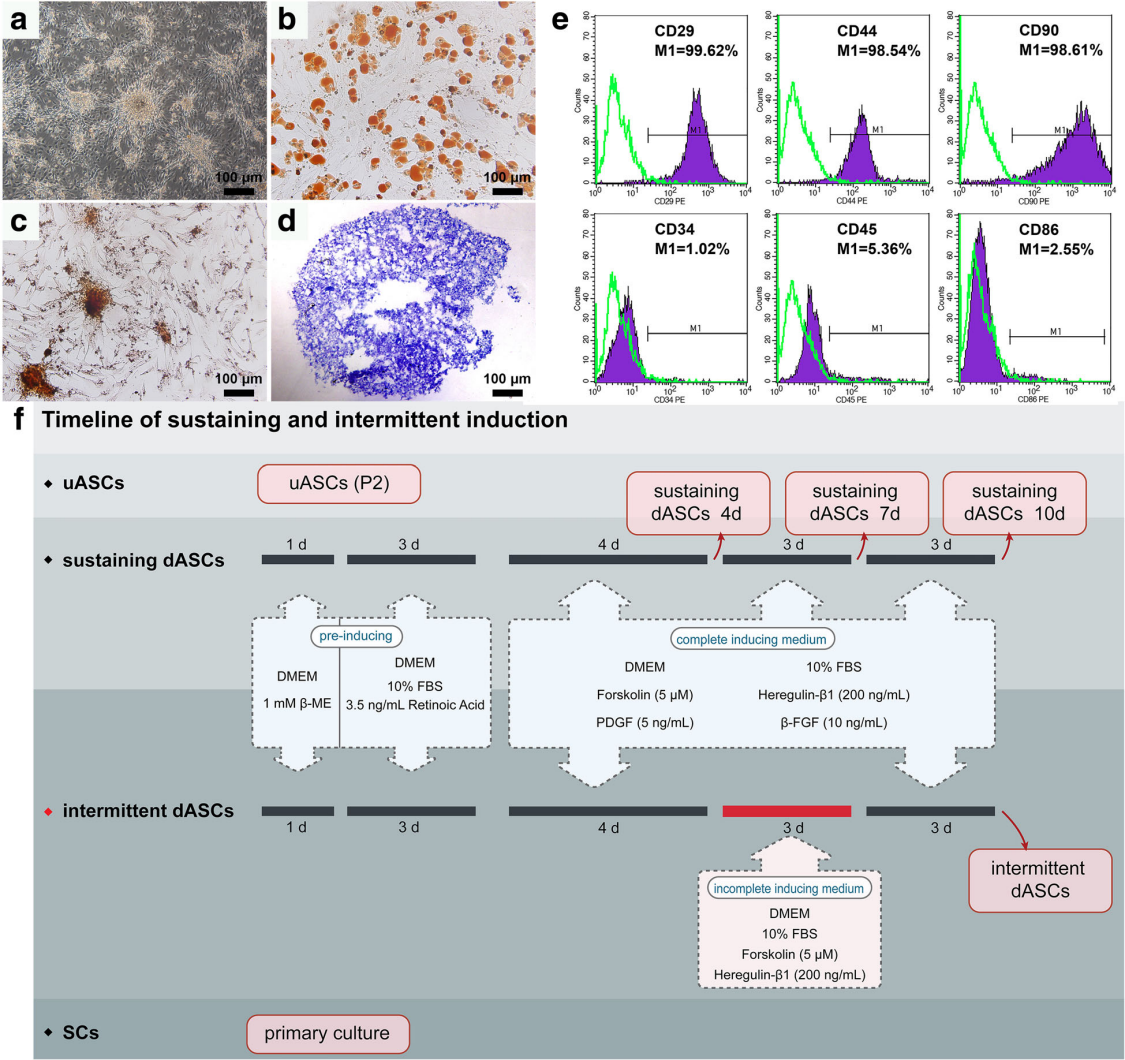
Identification of adipose-derived stem cells (ASCs) and schematic of the experimental design. **a** Primary ASCs grew in clusters and had a rounded spindle-like shape. **b** ASCs at passage 2 could differentiate into adipocytes, and the visual field in photographs was filled with red lipid droplets stained with Oil Red O solution. **c** ASCs differentiated into osteocytes formed calcium nodules with burrs, which were stained red by Alizarin Red solution. **d** In cartilage pellets, many cartilage lacunae were found, and the glycosaminoglycans around the chondrocytes were stained purple-blue by Toluidine Blue O solution. **e** Flow cytometry showed that more than 98% of ASCs were immunopositive for the MSC markers CD29, CD44, and CD90, while less than 6% of ASCs were immunopositive for the hematopoietic stem cell markers CD34, CD45, and CD86. **f** Schematic showing the experimental groups and induction methods. bFGF, basic fibroblast growth factor; β-ME, β-mercaptoethanol; dASC, differentiated ASC; DMEM, Dulbecco’s modified Eagle’s medium; FBS, fetal bovine serum; PDGF, platelet-derived growth factor; SC, Schwann cell; uASC, undifferentiated ASC

In this study, we improved the traditional methods for inducing the differentiation of MSCs into SCLCs. The experimental grouping and specific induction methods were as follows (Fig. 1f): (i) uASCs: undifferentiated ASCs (uASCs) at passage 2 were used; (ii–v) ASCs at passage 2 were pre-induced for 24 h with pre-induction medium I consisting of serum-free Dulbecco’s modified Eagle’s medium (DMEM) containing 1 mM β-mercaptoethanol (β-ME) (M3148; Sigma). Pre-induction medium I was replaced with pre-induction medium II consisting of DMEM, 10% FBS, and 35 ng/ml all *trans*-retinoic acid (RA) (R2625; Sigma) and incubated for 72 h. The resulting differentiated ASCs were divided into four groups according to subsequent processing: (ii) sustaining dASCs 4d: Differentiated ASCs (dASCs) were induced for 4 days with complete induction medium consisting of DMEM, 10% FBS, 5 μM forskolin (F6886; Sigma), 200 ng/ml recombinant human heregulin-β1 (HRG) (100–03; PeproTech), 10 ng/ml basic fibroblast growth factor (bFGF) (3339-FB: R&D Systems), and 5 ng/ml recombinant rat platelet-derived growth factor (PDGF)-AB (1115-AB; R&D Systems); (iii) sustaining dASCs 7d: dASCs were induced for 7 days with complete induction medium; (iv) sustaining dASCs 10d: dASCs were induced for 10 days with complete induction medium; (v) intermittent dASCs: dASCs were induced for 4 days with complete induction medium, after which complete induction medium was replaced with incomplete induction medium consisting of DMEM, 10% FBS, 5 μM forskolin, and 200 ng/ml HRG for induction for 3 days. Immediately after this procedure, complete induction medium was again used to induce differentiation for 3 days; and (vi) SCs: primary Schwann cells.

### Quantitative reverse transcription polymerase chain reaction (qRT-PCR)

Aliquots of more than 1 × 10^6^ cells were used to determine the specific gene expression profiles of cells in each group. Total RNA was extracted using an RNeasy Mini Kit (74,104; Qiagen) and quantified using a Nucleic Acid and Protein Analyzer (Microfuge18; Beckman-Coulter). The extracted RNA was reverse transcribed into complementary DNA (cDNA) using a ReverTra Ace® qPCR RT Kit (FSQ-101; TOYOBO). qRT-PCR was performed using a StepOne™ Real-Time PCR System (Applied Biosystems). Briefly, 1 μl of cDNA and 1 μl of specific primer pairs (Table 1) were used for qRT-PCR with DNA polymerase from the SYBR® Green Realtime PCR Master Mix-Plus (QPK-212; TOYOBO). The PCR amplification reaction was performed in a final volume of 20 μl under the following conditions: 1 min at 95 °C for enzyme activation, followed by 40 cycles of 15 s at 95 °C for denaturation, 15 s at 57 °C for annealing, and 45 s at 72 °C for extension. All data were analyzed using StepOne™ software version 2.2 (Applied Biosystems). Relative levels of gene expression were normalized to the level of the housekeeping gene, glyceraldehyde 3-phosphate dehydrogenase (GAPDH), and expressed as a ratio relative to uASCs. All detections were repeated three times independently.

**Table 1:** List of primer sequences used in this study

Target genes	Forward primer (5’–3’)	Reverse primer(5’–3’)	Annealing temperature (°C)
GAPDH	ATGGTGAAGGTCGGTGTGAACG	TTACTCCTTGGAGGCCATGTAG	55–62
P0	GCTCTTCTCTTCTTTGGTGCTGTCC	GGCGTCTGCCGCCCGCGCTTCG	57
NGFR p75	ATGAGGAGGGCAGGTGCTG	TCACACTGGGGACGTGGC	55
GFAP	GGACATCGAGATCGCCACCTACAG	CATCCCGCATCTCCACCGTCTTTAC	57
NGF	ATGTCCATGTTGTTCTACAC	TCAGCCTCTTCTTGCAGCC	56
BDNF	ATGACCATCCTTTTCCTTAC	CTATCTTCCCCTTTTAATGG	57
CNTF	ATGGCTTTCGCAGAGCAAA	CTACATCTGCTTATCTTTGGCC	56

BDNF, brain-derived neurotrophic factor; CNTF, ciliary neurotrophic factor; GAPDH, glyceraldehyde 3-phosphate dehydrogenase; GFAP, glial fibrillary acidic protein; NGF, nerve growth factor; NGFR p75, nerve growth factor receptor p75; P0, myelin protein zero

### Enzyme-linked immunosorbent assay (ELISA)

Cells in each group were resuspended in DMEM/F-12 containing 5% FBS and seeded in 25-cm^2^ cell culture flasks at a density of 3 × 10^4^ cells/cm^2^. The medium was collected and centrifuged at 1500 rpm at 4 °C for 10 min after 48 h of incubation. The concentrations of nerve growth factor (NGF), brain-derived neurotrophic factor (BDNF), glial cell line-derived neurotrophic factor (GDNF), and ciliary neurotrophic factor (CNTF) in the supernatant of each group were examined by ELISA using Rat NGF/NGF beta ELISA Kit (EK0471; Boster), Rat BDNF ELISA Kit (EK0308; Boster), Rat GDNF ELISA Kit (EK0363; Boster), and Rat CNTF ELISA Kit (EK0324; Boster), respectively. The absorbances of each well at 450 nm were determined using a trace orifice spectrophotometer (EPOCH TAKE 3; BioTek). The standard curve was drawn using the data produced from the diluted standard solutions with CurveExpert version 1.4. The concentrations of NGF, BDNF, GDNF, and CNTF were calculated according to the standard curve. All detections were repeated four times independently.

### Isolation of dorsal root ganglion and co-culture with uASCs/dASCs/SCs in a Transwell® system

Briefly, after sterilizing 12-h-old suckling SD rats in 75% ethanol solution, the spine was completely removed, and the blood was washed away with PBS. The spine was then dissected into two halves along the mid-sagittal plane, and the dorsal root ganglia (DRG) were removed from the ambilateral intervertebral foramen under a microscope. The epineurium of the DRG was carefully stripped using microforceps in cryic DMEM/F-12 medium supplemented with 10% FBS. DRG stripped from the epineurium were seeded in the lower chambers of a Transwell® system (3450; Corning) with six inserts, 24 mm in diameter with 0.4-μm pore size polyester membrane in six-well plates (Additional file 1: Figure S1). The six groups of cells were resuspended and seeded in the upper inserts of the Transwell® system at a density of 3 × 10^4^ cells/cm^2^ (Additional file 1: Figure S1). Neurobasal®-A Medium (10888–022; Gibco) supplemented with B-27® Serum-Free Supplement (1:50; 17,504–044; Gibco) and GlutaMAX™ Supplement (1:100; 35,050–061; Gibco) was used to co-culture the six groups of cells and DRG. After co-culture for 3 days, immunofluorescence staining of neurofilament protein 200 (NF200) and S100 was performed to evaluate the effects of each group of cells acting on the DRG. See the ***Immunofluorescence*** section below for detailed staining steps. Five randomly selected DRG from each group were imaged by integral photography. The three longest axons per quadrant (a total of four quadrants) were measured and used to calculate the mean axons length of each DRG using Image Pro Plus 6.0 (IPP 6.0; Media Cybernetics) image analysis software, as well as the farthest migration distance of SCs by calculating the distance between the three farthest SCs per quadrant and the DRG border. In addition, the ratio of S100-positive cells in each randomly selected DRG area was determined using IPP 6.0.

### Immunofluorescence

DRG co-cultured with six groups of cells in the Transwell® system and six groups of cells not from the Transwell® system were rinsed with PBS and fixed in 4% paraformaldehyde for 10 min at room temperature after removing the medium. Subsequently, the DRG and cells were blocked at room temperature with 10% goat serum (SL038; Solarbio) in PBS for 1 h after washing three times with PBS for 5 min each time. Mouse anti-S100 (1:1000; S2532; Sigma) and Rabbit anti-p75 NGF Receptor antibodies (1:500; ab8874; Abcam) were used as the primary antibodies for the six groups of cells. Mouse anti-Neurofilament 200 (1:800; N0142; Sigma) and Rabbit anti-S100 antibodies (1: 200; S2644; Sigma) were applied as the primary antibodies for DRG. After incubation with the respective primary antibodies in a humidified chamber overnight at 4 °C, DRG and cells were rinsed three times with PBS. Subsequently, Goat Anti-Mouse IgG H&L (Alexa Fluor® 488; 1:200; ab150117; Abcam) and Goat Anti-Rabbit IgG H&L (Alexa Fluor® 594; 1:200; ab150084; Abcam) secondary antibodies were applied and incubated with DRG and cells in the dark for 1 h at room temperature. Finally, the nuclei were counterstained with 4’,6-diamidino-2-phenylindole (DAPI) after washing three times with PBS. All images were captured using a microscope equipped with a DP71 camera. The number of surviving cells, and the rates of S100- and NGFR p75-positive cells in the six groups of cells (not from the Transwell® system) were calculated according to 10 randomly selected fields of each group at 200× magnification using IPP 6.0.

### Cell transplantation for the treatment of peripheral nerve defects

Fifty male SD rats, 12 weeks old and weighing 300–350 g, were anesthetized by intraperitoneal injection of 3% sodium pentobarbital solution (30 mg/kg body weight), and the hair on the left thigh was removed. The posterolateral skin of the left thigh was sterilized and incised. The sciatic nerve was carefully exposed and isolated from the intermuscular space. A 7-mm segment of the sciatic nerve was transected and removed using sharp microsurgery scissors, leaving a 10-mm defect after retraction of the nerve stumps. The rats were randomly separated into five groups (*n* = 10/group) according to the different types of nerve graft used to bridge the sciatic nerve defect: (A) hollow, in which only a biodegradable chitin conduit 10 mm in length (outer diameter, 2 mm; internal diameter, 0.8 mm) was used; (B) uASCs; (C) sustaining dASCs 7d, in which the differentiation effect was greatest among the sustaining induction groups in vitro; (D) intermittent dASCs; and (E) autograft, in which an autologous nerve graft was applied. In all groups involving cell transplantation, the biodegradable chitin conduits were injected with 10 μl of a mixture of fibrin glue and 5 × 10^5^ cells to construct different nerve grafts. Epineurial sutures were used to secure nerve grafts using an 8/0-swaged needle under an operating microscope. All rats were reared under standard laboratory conditions as described previously. The chitin conduits were provided by the Department of Orthopaedics and Trauma, Peking University People’s Hospital, and were patented by Peking University People’s Hospital and Textile Science Institute of China (Patent Number: 01136314.2).

### Gait analysis

Gait analysis was performed 2, 4, 6, 8, 10, and 12 weeks after transplantation to assess the recovery of motor function. A CatWalk footprint analysis system (XT 10.6; Noldus) was used to collect footprints, and the assessment process was as follows. Rats in each group were placed on the walkway floor; when the paws touched the glass floor, the dynamic properties changed due to the pressure, and fluorescence escaped internal reflectance. Thus, only the contact area of the paws was visible and the footprint appeared brighter when the paws provided more pressure. When the rat entered a 40 × 10 cm field on the walkway, the run was captured by a video camera underneath the walkway floor. The run was approved if the rat passed the monitored field within 5 s and the speed showed less than 50% variation. At least three approved runs were needed for each group per time point for the analysis. All acquired data were processed in a semiautomated manner using the Noldus Catwalk analysis software, which required manual definition of the limb (LH = left hind, RH = right hind) from the recorded run. The sciatic functional index (SFI) and stand/swing time ratio of each rat were calculated using CatWalk XT 10.6 according to the measurement results.

### Electrophysiology

Neural electrophysiological evaluation was performed at the twelfth week after transplantation. Five rats in each group were anesthetized with 3% sodium pentobarbital solution. The previous surgical site on the left femur was re-opened, and the postoperative sciatic nerves and gastrocnemius of each rat were exposed. The compound muscle action potentials (CMAPs) were induced and recorded using a fully functional electromyography machine (Keypoint; Medtronic), the bipolar stimulating electrodes of which were placed sequentially on the proximal and distal ends of each graft and the monopolar recording electrode was placed on the belly of the ipsilateral gastrocnemius. The CMAPs of the contralateral normal sciatic nerve were also recorded for normalization. Electrical stimulation was performed at 3.0 mA and 1 Hz. The peak amplitude and latency of CMAPs were used to evaluate the recovery of nerve conduction function, and the acquired data were expressed as the ratio of the injured side to the normal side.

### Histological evaluation of grafting segment following cell transplantation

Five rats in each group were sacrificed by intraperitoneal injection of a sodium pentobarbital overdose in week 6 following cell transplantation. The implanted nerve grafts were clearly exposed and integrally excised. Cross-sections of each nerve graft midpoint were cut into 7-μm thick paraffin sections and placed in Sodium Citrate Antigen Retrieval Solution (C1032; Solarbio) for 10 min at 95 °C after deparaffinization. The sections were then transferred to PBS containing 10% goat serum to block heterogenetic antigens for 30 min at room temperature after washing three times with PBS for 5 min each time. The primary antibodies Rabbit anti-myelin protein zero (P0) (1: 100; ab31851; Abcam) and Mouse anti-NF200 (1:800; N0142; Sigma) were used to incubate sections in a humidified chamber overnight at 4 °C. Next morning, Goat Anti-Mouse and Goat Anti-Rabbit IgG H&L secondary antibodies were applied to the sections and incubated in the dark for 1 h at room temperature after rinsing off excess primary antibodies with PBS. After washing three times with PBS, the nuclei were counterstained with DAPI. Ten randomly selected fields of each group at 400× magnification were captured to count the mean density of myelinated nerve fibers by IPP 6.0.

At week 12 after transplantation, the rats were sacrificed by intraperitoneal injection of an overdose of sodium pentobarbital following electrophysiological evaluation. The regenerative nerves located at the distal ends of the nerve grafts were fixed to cut transverse semi-thin sections at a thickness of 1 μm using a glass knife in an ultramicrotome (EM UC7; Leica) and stained with Toluidine Blue O solution (1% in sodium borate; G3663; Solarbio). The stained sections were observed under 400× magnification, and 10 randomly selected fields were captured for each group to count the mean density of myelinated nerve fibers by IPP 6.0. Subsequently, the regenerative nerves were cut into transverse ultrathin sections 70 nm thick using a diamond knife in an ultramicrotome (EM UC7; Leica) and collected on copper slot grids with pioloform/carbon support films. The ultrathin sections were counterstained with 3% lead citrate and uranyl acetate, and were observed by transmission electron microscopy (TEM) (CM-120; Philips). Ten randomly selected fields at 5000× magnification were captured for each group to measure the mean diameter of myelinated nerve fibers and the mean thickness of the myelin sheath by IPP 6.0.

### Ultrasonic and histological evaluation of the gastrocnemius

Ultrasonic elastography was used to evaluate the muscle activity of the gastrocnemius at the sixth week after transplantation. Briefly, rats from each group were anesthetized with 3% sodium pentobarbital solution (30 mg/kg body weight) and the hair was removed from the paired hind limbs. The body surface projective regions of the bilateral gastrocnemius were fully exposed. A color Doppler ultrasound diagnostic instrument (Apolio500; TOSHIBA) was applied to locate the gastrocnemius using two-dimensional imaging. Subsequently, the instrument was switched to the shear wave elastography (SWE) mode, and the sampling frame was adjusted using the continuous excitation mode without pressing on the ultrasonic probe. The image was frozen and regulated to the speed propagation pattern after it had stabilized. Images with good isochrone were selected, and the area of interest was selected using the acquiescent circle to automatically record Young’s modulus and standard deviation, which were used for statistical analyses.

At the twelfth week after transplantation, the bilateral gastrocnemius muscles of rats in each group were excised and immediately weighed to calculate the wet weight recovery ratio of the gastrocnemius (injured side/normal side) after electrophysiological evaluation. The gastrocnemius muscle belly was harvested and fixed in 4% formaldehyde for 2 h. Transverse paraffin sections of the gastrocnemius muscle belly 10 μm thick were cut and stained using a modified Masson’s trichrome stain kit (G1345; Solarbio). Ten randomly selected fields in each group at 200× magnification were captured to measure the mean cross-sectional area of the gastrocnemius fibers by IPP 6.0.

### Statistical analysis

All data are presented as the means ± standard deviation (SD) from three or more independent experiments. Statistical analysis was performed using SPSS (version 22.0; IBM). Student’s *t* test was performed to compare differences between two groups, and one-way analysis of variance (ANOVA) was used to compare differences between multiple groups. Tukey’s post-hoc test was applied when *p* > 0.05 in the test of homogeneity of variances, otherwise Dunnett’s T3 post-hoc test was applied. Differences between groups were considered significant at **p < 0.01 and *p < 0.05.

## Results

### Identification of adipose-derived stem cells

Primary ASCs grew in clusters and had a rounded spindle-like shape (Fig. 1a). ASCs at passage 2 could differentiate into adipocytes, and the visual field in photographs was filled with red lipid droplets stained with Oil Red O solution (Fig. 1b). Upon osteogenic differentiation, differentiated ASCs showed calcium nodule deposition with burrs, which stained positively with Alizarin Red solution (Fig. 1c). Many cartilage lacunae were found in cartilage pellets, which were induced from ASCs. In addition, glycosaminoglycans around the chondrocytes induced from ASCs were stained purple-blue by Toluidine Blue O solution (Fig. 1d). Flow cytometry showed that more than 98% of ASCs were immunopositive for the MSC markers CD29, CD44, and CD90, while less than 6% of ASCs were immunopositive for the hematopoietic stem cell markers CD34, CD45, and CD86 (Fig. 1e).

### Characteristics of Schwann cell-like cells

**Fig. 2:**
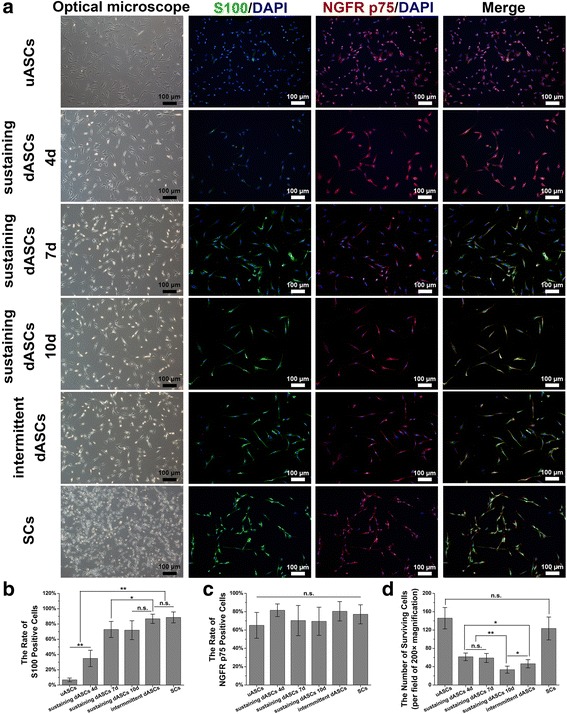
Characteristics of Schwann cell (SC)-like cells derived from differentiated adipose-derived stem cells (dASCs) by sustaining/intermittent induction methods. **a** In the leftmost column of optical microscope photographs, undifferentiated ASCs (uASCs) had a spindle-like shape and, with extension of the induction period, the dASCs became morphologically more similar to SCs, exhibiting a narrow fusiform-like shape with a bipolar or tripolar structure. In the immunofluorescence photographs, in the second column, S100-positive cells are stained with green fluorescence; nerve growth factor receptor (NGFR) (p75)-positive cells are stained with red fluorescence in the third column. The blue fluorescence always corresponds to cell nuclei. In the rightmost column of the immunofluorescence photographs, the three areas of fluorescence merge. **b** The rate of S100-positive cells in intermittent dASCs group (86.78 ± 6.03%) was not significantly different compare to that in sustaining dASCs 10d (71.89 ± 12.37%) or SCs groups (88.60 ± 7.15%), but was significantly higher (*p* < 0.05) than the uASCs (6.80 ± 2.44%), sustaining dASCs 4d (34.90 ± 10.70%), and sustaining dASCs 7d groups (72.81 ± 10.53%). **c** The rate of NGFR (p75)-positive cells was not significantly different among all six groups. **d** The number of surviving cells in sustaining dASCs 10d (33.50 ± 7.74) was significantly less (*p* < 0.05) than that in intermittent dASCs (46.40 ± 9.00), sustaining dASCs 4d (61.50 ± 8.32), and 7d groups (59.00 ± 9.85). Data are expressed as means ± SEM. *p < 0.05, **p < 0.01, one-way ANOVA with Tukey’s post-test or Dunnett’s T3 post-test. n.s., no significant difference

uASCs had a spindle-like shape, although very few expressed S100 protein, an important marker of SCs, and more than 60% were immunopositive for NGFR p75 (Fig. 2a–c). These results indicated that ASCs had the potential to differentiate into glial cells. With extension of the induction period, the dASCs became morphologically more similar to SCs, exhibiting a narrow fusiform-like shape with a bipolar or tripolar structure, and the rate of S100-positive cells labeled with green fluorescence also increased gradually (Fig. 2a, b). However, the number of surviving cells in each sustaining dASC group decreased gradually with induction time due to apoptosis of unsuccessfully differentiated ASCs, which indirectly increased the rate of S100-positive cells. Among them, the number of surviving cells in sustaining dASCs 10d (33.50 ± 7.74) was significantly less (*p* < 0.05) than that in intermittent dASCs (46.40 ± 9.00), sustaining dASCs 4d (61.50 ± 8.32), and sustaining dASCs 7d groups (59.00 ± 9.85) (Fig. 2a, d). In the meantime, the rate of S100-positive cells in the intermittent dASCs group (86.78 ± 6.03%) was not significantly different compared with that in sustaining dASCs 10d (71.89 ± 12.37%) or SCs groups (88.60 ± 7.15%), but was significantly higher (*p* < 0.05) than the uASCs (6.80 ± 2.44%), sustaining dASCs 4d (34.90 ± 10.70%), and sustaining dASCs 7d groups (72.81 ± 10.53%) (Fig. 2a, b). In addition, the rate of NGFR p75-positive cells was not significantly different among all six groups (Fig. 2a, c).

### mRNA expression

**Fig. 3:**
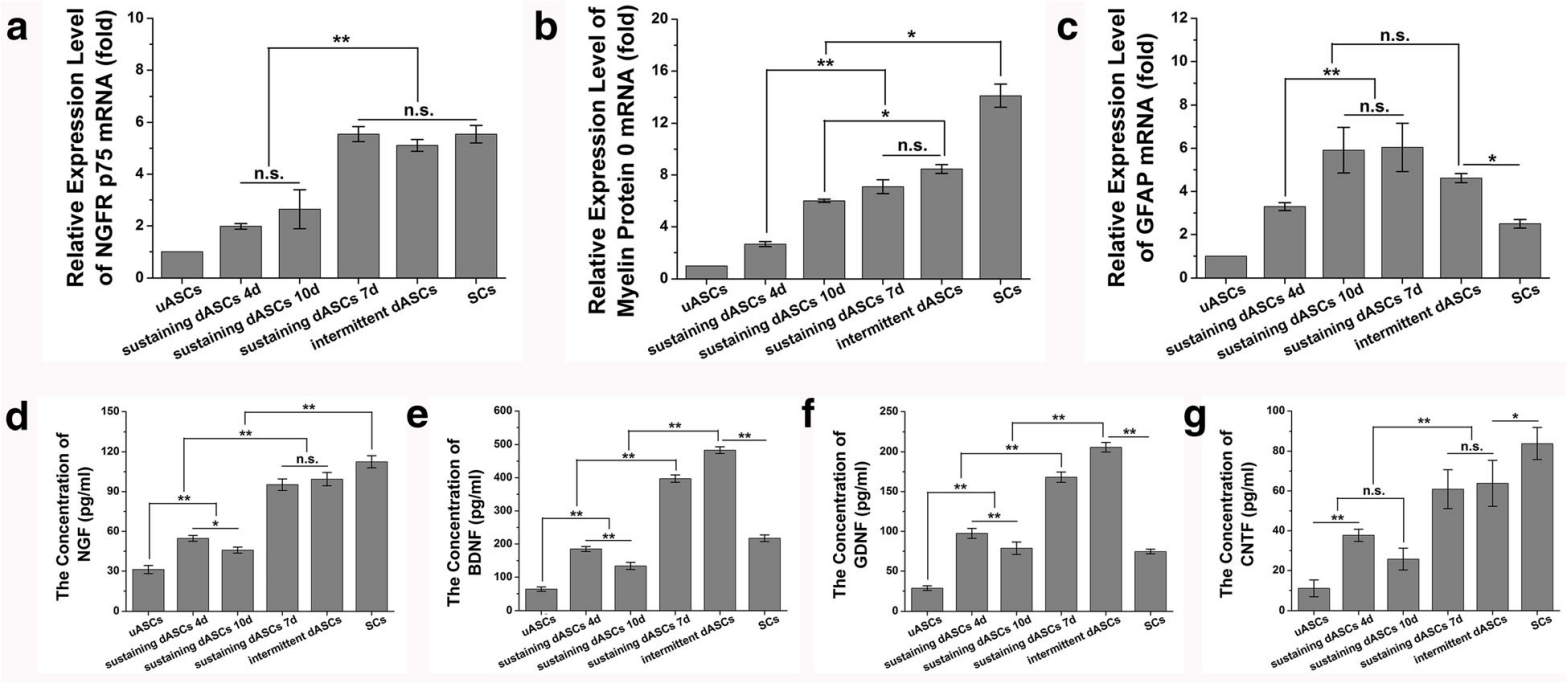
Relative messenger RNA (mRNA) expression and neurotrophin secretion levels of all groups. **a** The relative expression level of nerve growth factor receptor (NGFR) (p75) mRNA was not significantly different among sustaining dASCs 7d (5.55 ± 0.29), intermittent dASCs (5.11 ± 0.23), and SCs (5.54 ± 0.34), which were significantly higher (*p* < 0.01) than the other three groups. **b** The relative expression level of P0 mRNA was highest in SCs (14.11 ± 0.88). There were no significant differences between intermittent dASCs (8.46 ± 0.34) and sustaining dASCs 7d (7.10 ± 0.54). However, intermittent dASCs showed significantly higher P0 mRNA expression (*p* < 0.05) in comparison with sustaining dASCs 10d (6.00 ± 0.12) and 4d groups (2.66 ± 0.19). **c** The relative expression level of glial fibrillary acidic protein (GFAP) mRNA was not significantly different between intermittent dASCs (4.62 ± 0.21) and the three sustaining dASCs groups, all of which showed significant upregulation (*p* < 0.01) in comparison with the undifferentiated adipose-derived stem cells (uASCs). **d** The levels of nerve growth factor (NGF) secreted by intermittent dASCs (99.37 ± 5.00 pg/ml) and sustaining dASCs 7d (95.20 ± 4.34 pg/ml) were significantly higher (*p* < 0.01) than those of uASCs (31.18 ± 3.09 pg/ml), and sustaining dASCs 4d (54.69 ± 2.20 pg/ml) and 10d (45.90 ± 2.27 pg/ml), but lower (*p* < 0.01) than that of SCs (112.46 ± 4.55 pg/ml). **e**,**f** Brain-derived neurotrophic factor (BDNF) and glial cell line-derived neurotrophic factor (GDNF) secretion levels showed similar tendencies. The concentrations of BDNF and GDNF secreted by intermittent dASCs were 483.01 ± 10.08 and 205.66 ± 6.01 pg/ml, respectively, which were higher (*p* < 0.01) than in the other groups, including SCs. **g** The concentrations tendency of ciliary neurotropic factor (CNTF) and NGF were similar. Data are expressed as means ± SEM. *p < 0.05, **p < 0.01, one-way ANOVA with Tukey’s post-test or Dunnett’s T3 post-test. dASC, differentiated adipose-derived stem cell; n.s., no significant difference, SC, Schwann cell

To compare the differentiation effect at the molecular level, the relative mRNA expression levels in each group of cells were examined by qRT-PCR. The relative expression level of NGFR p75 mRNA was not significantly different among sustaining dASCs 7d (5.55 ± 0.29), intermittent dASCs (5.11 ± 0.23), and SCs (5.54 ± 0.34), which were significantly higher (*p* < 0.01) than the other three groups (Fig. 3a). The relative expression level of P0 mRNA was highest in SCs (14.11 ± 0.88). There were no significant differences between intermittent dASCs (8.46 ± 0.34) and sustaining dASCs 7d (7.10 ± 0.54). However, intermittent dASCs showed significantly higher (*p* < 0.05) P0 mRNA expression in comparison with sustaining dASCs 10d (6.00 ± 0.12) and sustaining dASCs 4d (2.66 ± 0.19) (Fig. 3b). The relative expression level of GFAP mRNA was not significantly different between intermittent dASCs (4.62 ± 0.21) and the three sustaining dASCs groups, all of which showed significant upregulation (*p* < 0.01) in comparison with the uASCs (Fig. 3c). The relative mRNA expression levels of neurotrophins, including NGF, BDNF, and CNTF, were also evaluated (Additional file 2: Figure S2). There were no significant differences in NGF, BDNF, or CNTF mRNA expression levels between intermittent dASCs and sustaining dASCs 7d, but all were significantly upregulated (*p* < 0.01) compared with sustaining dASCs 4d and sustaining dASCs 10d (Additional file 2: Figure S2a–c).

### Secretion of neurotrophins

The neurotrophins secreted by the six groups of cells were measured at the protein level by ELISA. The levels of NGF secreted by intermittent dASCs (99.37 ± 5.00 pg/ml) and sustaining dASCs 7d (95.20 ± 4.34 pg/ml) were significantly higher (*p* < 0.01) than those of uASCs (31.18 ± 3.09 pg/ml) and sustaining dASCs 4d (54.69 ± 2.20 pg/ml) and sustaining dASCs 10d (45.90 ± 2.27 pg/ml), but were lower (*p* < 0.01) than that of SCs (112.46 ± 4.55 pg/ml) (Fig. 3d). A similar tendency was found for CNTF (Fig. 3g). In addition, BDNF and GDNF secretion levels also showed similar tendencies. The concentrations of BDNF and GDNF secreted by intermittent dASCs were 483.01 ± 10.08 and 205.66 ± 6.01 pg/ml, respectively, which were higher (*p* < 0.01) than in the other groups, including SCs (Fig. 3e, f).

### Schwann cell-like cells mediated trophic effect on DRG

**Fig. 4:**
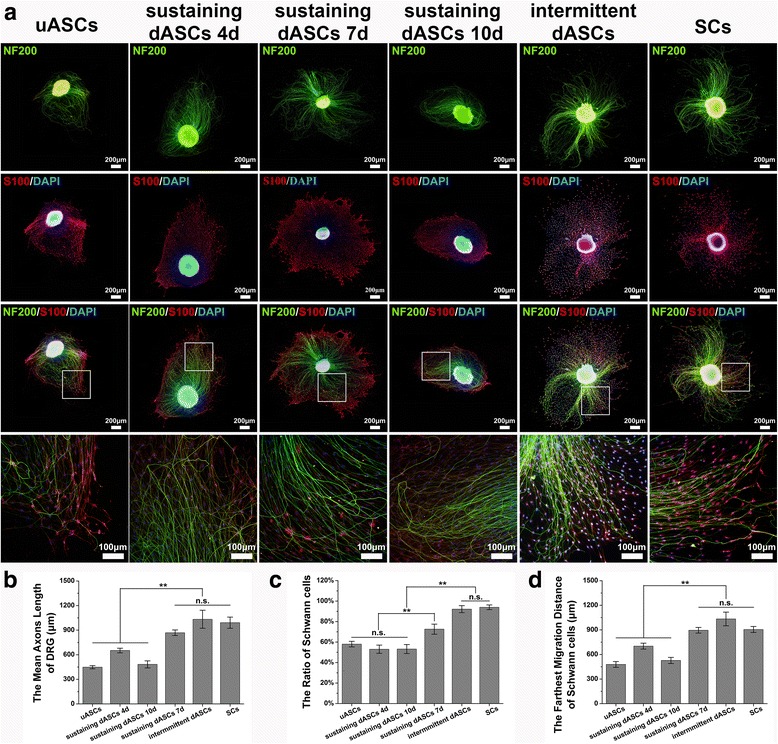
Effects of the secretions of each group of cells on dorsal root ganglia (DRG). **a** The axons of DRG exhibited green fluorescence and Schwann cells (SCs) that migrated from the DRG exhibited red fluorescence in the lower chamber of the Transwell® system. Simultaneously, all nuclei showed blue fluorescence. Moreover, the distributions of cells in undifferentiated adipose-derived stem cells (uASCs) and all sustaining differentiated (d)ASCs groups were serried, whereas those in intermittent dASCs and SCs were homogeneous. Photographs in the bottom row are higher magnification views of those in the box in the third row. **b** The mean axon length of DRG in sustaining dASCs 7d (868.53 ± 34.22 μm), intermittent dASCs (1032.77 ± 109.91 μm), and SCs (991.41 ± 68.23 μm) were significantly higher (*p* < 0.01) than those in the other three groups, but there were no significant differences within the three groups. **c** Although the ratio of SCs in sustaining dASCs 7d (72.62 ± 4.92%) was significantly higher (*p* < 0.01) than those in uASCs (58.01 ± 2.74%), and sustaining dASCs 4d (53.02 ± 4.03%) and 10d (53.22 ± 4.38%), the ratios in the above four groups were significantly lower (*p* < 0.01) than in those in intermittent dASCs (92.14 ± 3.44%) and SCs (93.95 ± 2.28%). **d** A similar tendency was observed for the mean axon length of DRG, as the SCs showed significantly greater (*p* < 0.01) migration distance from the DRG in sustaining dASCs 7d (895.38 ± 34.80 μm), intermittent dASCs (1033.19 ± 83.56 μm), and SCs (904.90 ± 36.83 μm) compared with the other three groups, although there were no significant differences between these three groups. Data are expressed as means ± SEM. **p < 0.01, one-way ANOVA with Tukey’s post-test or Dunnett’s T3 post-test. n.s., no significant difference,

Immunofluorescence analysis revealed that the axons of DRG exhibited green fluorescence, and SCs that migrated from the DRG exhibited red fluorescence in the lower chamber of the Transwell® system. Simultaneously, all nuclei showed blue fluorescence (Fig. 4a). The mean axon length of DRG in sustaining dASCs 7d (868.53 ± 34.22 μm), intermittent dASCs (1032.77 ± 109.91 μm), and SCs (991.41 ± 68.23 μm) were significantly higher (*p* < 0.01) than those in the other three groups, but there were no significant differences within these three groups (Fig. 4b). Although the ratio of SCs in sustaining dASCs 7d (72.62 ± 4.92%) was significantly higher (*p* < 0.01) than those in uASCs (58.01 ± 2.74%), and sustaining dASCs 4d (53.02 ± 4.03%) and sustaining dASCs 10d (53.22 ± 4.38%), the ratios in the above four groups were significantly lower (*p* < 0.01) than in those in intermittent dASCs (92.14 ± 3.44%) and SCs (93.95 ± 2.28%). Moreover, the distributions of cells in uASCs and all sustaining dASCs groups were serried, whereas those in intermittent dASCs and SCs were homogeneous (Fig. 4a, c). A similar tendency was observed for the mean axon length of DRG, as the SCs showed significantly greater (*p* < 0.01) migration distance from the DRG in sustaining dASCs 7d (895.38 ± 34.80 μm), intermittent dASCs (1033.19 ± 83.56 μm), and SCs (904.90 ± 36.83 μm) compared with the other three groups, although there were no significant differences between these three groups (Fig. 4d).

### Functional recovery of the sciatic nerve following cell transplantation

**Fig. 5:**
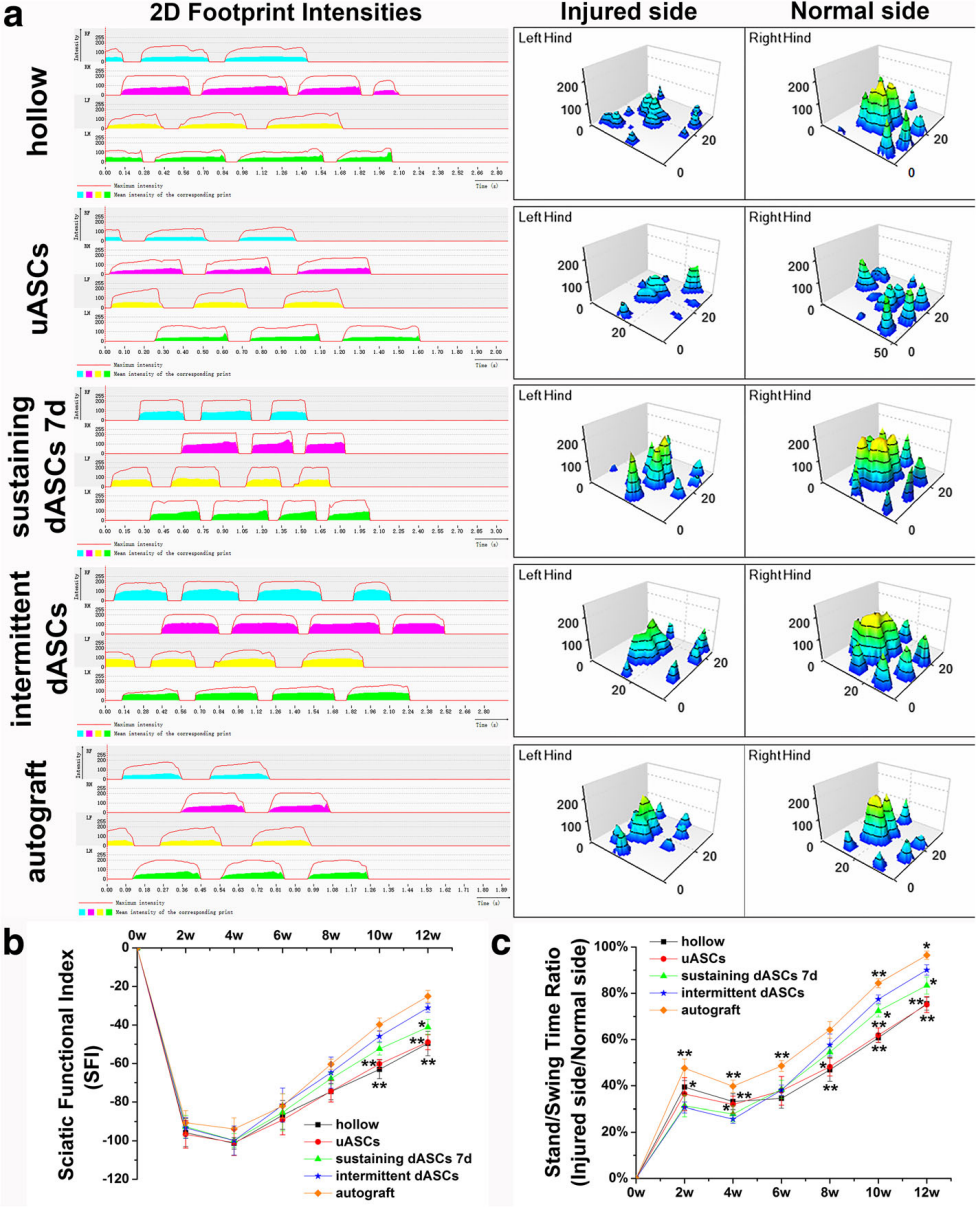
Gait analysis and sciatic functional recovery after cell transplantation. **a** Representative two-dimensional (2D) and three-dimensional (3D) stress diagrams of each group at 12 weeks after cell transplantation. **b** The obvious advantage began to appear in the SFI in the intermittent dASCs group at week 10 following cell transplantation. The improvement of the SFI in the intermittent dASCs group (−31.07 ± 2.24) was significantly superior (*p* < 0.05) to those in the hollow (−49.58 ± 6.35), uASCs (−48.88 ± 3.93), and sustaining dASCs 7d groups (−41.14 ± 4.03) in the twelfth week after cell transplantation, and showed no significant difference compared with the autograft group (−25.09 ± 3.13). **c** In the first 4 weeks, the stand/swing time ratio in intermittent dASCs was always significantly lower (*p* < 0.05) than those in the hollow, uASCs, and autograft groups. The advantages of the intermittent dASCs group began to appear at week 8 after cell transplantation. Until the last time point, the stand/swing time ratio in the intermittent dASCs (90.10 ± 2.25%) was significantly better (*p* < 0.05) than those in the hollow (75.51 ± 2.80%), uASCs (75.13 ± 3.47%), and sustaining dASCs 7d groups (83.39 ± 3.68%). Data are expressed as means ± SEM. *p < 0.05, **p < 0.01, one-way ANOVA with Tukey’s post-test or Dunnett’s T3 post-test. dASC, differentiated adipose-derived stem cell; uASC, undifferentiated adipose-derived stem cell

Functional recovery of the sciatic nerve was evaluated by gait analysis. Representative two-dimensional (2D) and three-dimensional (3D) stress diagrams of each group at 12 weeks after cell transplantation are shown in Fig. 5a. According to the 2D stress diagrams, the period of contact with the floor was markedly shorter for the left hind (LH) leg on the injured side compared with the normal right hind (RH) leg in hollow and uASCs groups, while the contact times in the sustaining dASCs 7d and intermittent dASCs groups were closer to that in the autograft group. Furthermore, the intensity of pressure with which the LH leg contacted the floor was markedly reduced in comparison with the RH leg in hollow and uASC groups, but there were no significant differences between the RH and LH legs in the sustaining dASCs 7d or intermittent dASCs groups, as well as the autograft group. Although the 3D stress diagrams showed less contact area of the LH leg than the RH leg, the length and width of the LH toes in sustaining dASCs 7d and intermittent dASCs were improved compared with the hollow and uASC groups, and were closer to those in the autograft group (Fig. 5a).

The obvious advantage began to appear in the SFI in the intermittent dASCs group at week 10 following cell transplantation. The improvement of the SFI in the intermittent dASCs group (−31.07 ± 2.24) was significantly superior (*p* < 0.05) to those in the hollow (−49.58 ± 6.35), uASCs (−48.88 ± 3.93), and sustaining dASCs 7d groups (−41.14 ± 4.03) in the twelfth week after cell transplantation, and showed no significant difference compared with the autograft group (−25.09 ± 3.13) (Fig. 5b).

In the first 4 weeks, the stand/swing time ratio in intermittent dASCs was always significantly lower (*p* < 0.05) than those in the hollow, uASCs, and autograft groups. The advantages of the intermittent dASCs group began to appear at week 8 after cell transplantation. Until the last time point, the stand/swing time ratio in the intermittent dASCs (90.10 ± 2.25%) was significantly better (*p* < 0.05) than those in the hollow (75.51 ± 2.80%), uASCs (75.13 ± 3.47%), and sustaining dASCs 7d groups (83.39 ± 3.68%) (Fig. 5c).

### Histological evaluation of regenerative nerves following cell transplantation

**Fig. 6:**
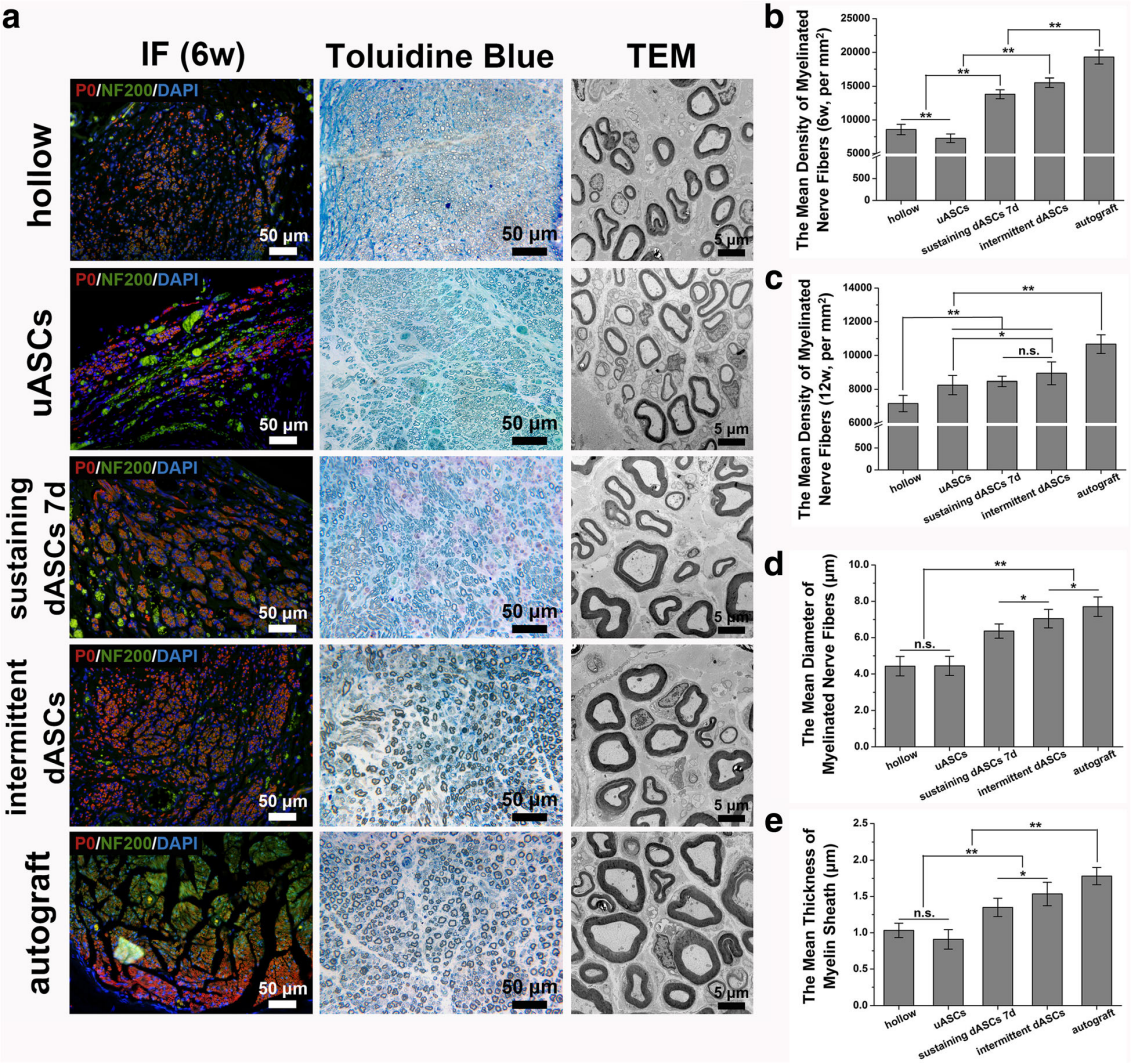
Histological evaluation of regenerated nerves after cell transplantation. **a** In the immunofluorescence (IF) photographs in the leftmost column, regenerated axons stained with green fluorescence (wrapping or not wrapping around regenerated myelin sheaths labeled with red fluorescence) were found at the midpoint of the implanted segments at week 6 after cell transplantation. The nuclei were stained with blue fluorescence. The photographs in the middle and rightmost columns show Toluidine Blue staining and transmission electron microscopy (TEM) images, respectively, of regenerated nerves located at the distal end of implanted segments at week 12 after cell transplantation. **b,c** The mean density of myelinated nerve fibers in intermittent dASCs (15,520 ± 694/mm^2^) was significantly greater (*p* < 0.01) than those in the hollow (8575 ± 779/mm^2^), uASCs (7268 ± 637/mm^2^), and sustaining dASCs 7d groups (13,814 ± 658/mm^2^) at week 6 after cell transplantation. However, no significant difference was observed compared with the sustaining dASCs 7d (8942 ± 670/mm^2^ vs. 8461 ± 301/mm^2^) at week 12 after cell transplantation. **d,e** The mean diameter of myelinated nerve fibers and mean thickness of myelin sheath showed similar trends, i.e., those in the intermittent dASCs (7.05 ± 0.51 μm in diameter and 1.53 ± 0.16 μm in thickness) were significantly greater (*p* < 0.05) than those in the hollow (4.43 ± 0.54 μm in diameter and 1.03 ± 0.10 μm in thickness), uASCs (4.44 ± 0.53 μm in diameter and 0.91 ± 0.14 μm in thickness), and sustaining dASCs 7d groups (6.36 ± 0.39 μm in diameter and 1.35 ± 0.13 μm in thickness), but significantly less (*p* < 0.05) than those in the autograft group (7.70 ± 0.53 μm in diameter and 1.78 ± 0.12 μm in thickness). Data are expressed as means ± SEM. *p < 0.05, **p < 0.01, one-way ANOVA with Tukey’s post-test or Dunnett’s T3 post-test. dASC, differentiated adipose-derived stem cell; n.s., no significant difference; uASC, undifferentiated adipose-derived stem cell

At week 6 after cell transplantation, regenerated axons labeled with green fluorescence with or without regenerated myelin sheaths labeled with red fluorescence were observed at the midpoint of the implanted segments. The nuclei were labeled with blue fluorescence (Fig. 6a). The mean density of myelinated nerve fibers in intermittent dASCs (15,520 ± 694/mm^2^) was significantly greater (*p* < 0.01) than those in the hollow (8575 ± 779/mm^2^), uASCs (7268 ± 637/mm^2^), and sustaining dASCs 7d groups (13,814 ± 658/mm^2^) at week 6 after cell transplantation. However, no significant difference was observed compared with the sustaining dASCs 7d group (8942 ± 670/mm^2^ vs. 8461 ± 301/mm^2^) at week 12 after cell transplantation (Fig. 6b, c).

At week 12 after cell transplantation, Toluidine Blue staining and TEM results of regenerative nerves located at the distal ends of implanted segments showed relatively tenuous myelinated nerve fibers, and thinner myelin sheaths were found in the hollow, uASCs, and sustaining dASCs 7d groups, while thicker myelinated nerve fibers and myelin sheaths were found in the intermittent dASCs and autograft groups. In the hollow, uASCs, and sustaining dASCs 7d groups, more fibrillar connective tissue was seen among the myelinated or non-myelinated nerve fibers, whereas little fibrillar connective tissue was found in the intermittent dASCs and autograft groups (Fig. 6a). The mean diameter of myelinated nerve fibers and mean thickness of myelin sheaths showed similar trends, i.e., those in the intermittent dASCs (7.05 ± 0.51 μm in diameter and 1.53 ± 0.16 μm in thickness) were significantly greater (*p* < 0.05) than those in the hollow (4.43 ± 0.54 μm in diameter and 1.03 ± 0.10 μm in thickness), uASCs (4.44 ± 0.53 μm in diameter and 0.91 ± 0.14 μm in thickness), and sustaining dASCs 7d groups (6.36 ± 0.39 μm in diameter and 1.35 ± 0.13 μm in thickness), but significantly less (*p* < 0.05) than those in the autograft group (7.70 ± 0.53 μm in diameter and 1.78 ± 0.12 μm in thickness) (Fig. 6d, e).

### Ultrasonic and histological evaluation of the gastrocnemius

**Fig. 7:**
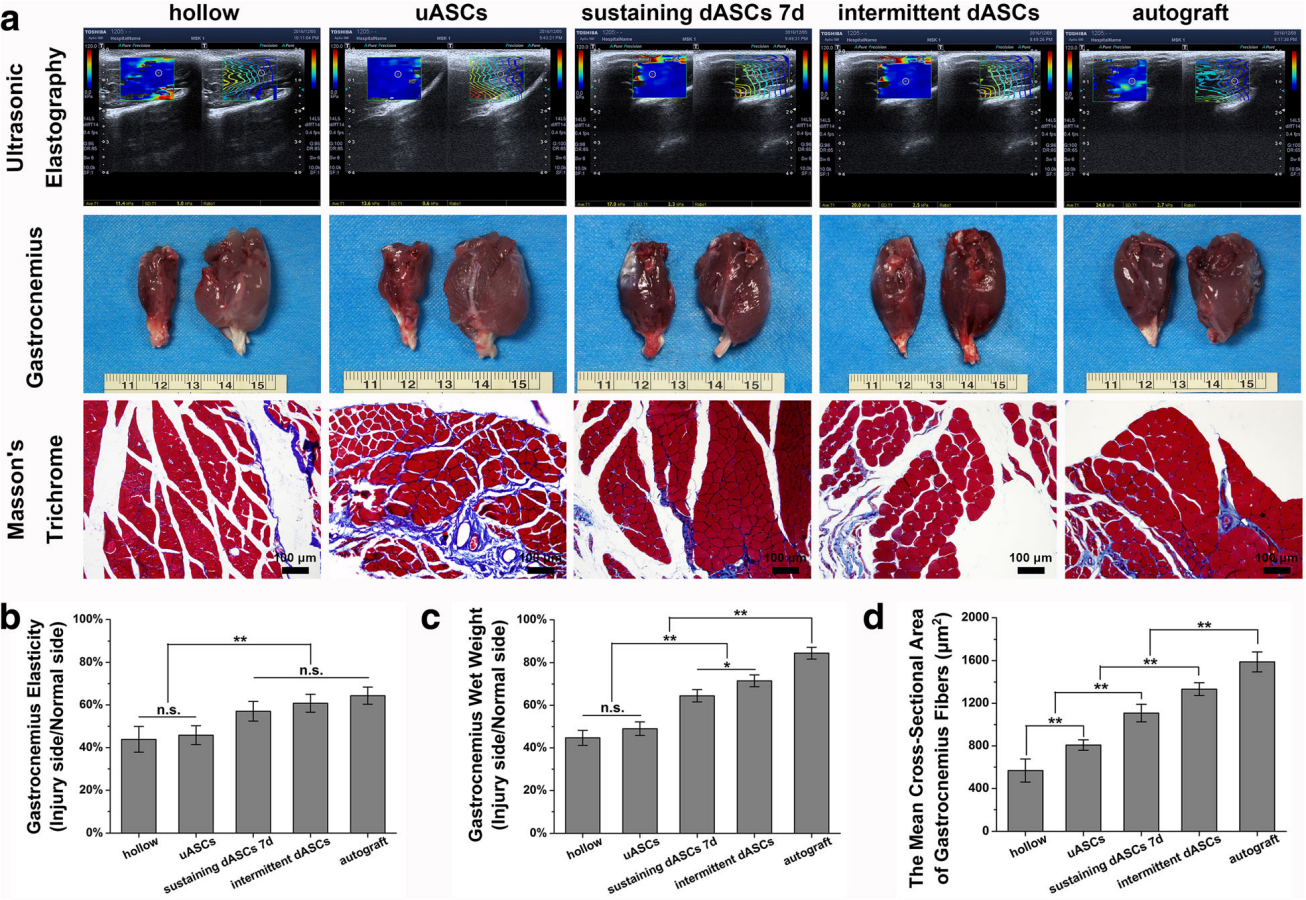
Ultrasonic and histological evaluation of the gastrocnemius. **a** Representative results of ultrasonic elastography of the gastrocnemius at week 6 after cell transplantation are shown in the first row. The elastomeric map (left) and Young’s modulus maps (right) are shown for each group. In the photographs in the middle row, the gastrocnemius on the injured side shows visible atrophy in the hollow and uASCs groups compared with the normal side at week 12 after cell transplantation; in contrast, the other three groups were less pronounced. Representative Masson’s trichrome staining results of the gastrocnemius are shown in the bottom row. All groups showed hyperplasia of blue-stained collagen fibers distributed among muscle fibers stained in red. **b** Although the gastrocnemius elasticity ratio was not significantly different among sustaining dASCs 7d (57.05 ± 4.59%), intermittent dASCs (60.79 ± 4.21%), and autograft groups (64.36 ± 4.01%), these values were significantly higher (*p* < 0.01) than those in the hollow (43.88 ± 6.03%) and uASCs groups (45.81 ± 4.44%). **c** At the twelfth week after cell transplantation, the gastrocnemius wet weight ratio in intermittent dASCs (71.46 ± 2.78%) was significantly greater (*p* < 0.05) than those in the hollow (44.70 ± 3.51%), uASCs (49.00 ± 3.15%), and sustaining dASCs 7d groups (64.45 ± 2.91%). **d** The mean cross-sectional area of gastrocnemius fibers in the intermittent dASCs group (1332.24 ± 60.17 μm^2^) was significantly greater (*p* < 0.01) than those in the hollow (568.04 ± 108.59 μm^2^), uASCs (807.80 ± 49.20 μm^2^), and sustaining dASCs 7d groups (1107.81 ± 82.86 μm^2^) at the twelfth week after cell transplantation. Data are expressed as means ± SEM. *p < 0.05, **p < 0.01, one-way ANOVA with Tukey’s post-test or Dunnett’s T3 post-test. dASC, differentiated adipose-derived stem cell; n.s., no significant difference; uASC, undifferentiated adipose-derived stem cell

Representative results of ultrasonic elastography of the gastrocnemius at week 6 after cell transplantation are shown in Fig. 7a to evaluate the target organ state of regenerative nerves. The elastomeric map (left) and Young’s modulus maps (right) are shown for each group. Although the gastrocnemius elasticity ratio was not significantly different among sustaining dASCs 7d (57.05 ± 4.59%), intermittent dASCs (60.79 ± 4.21%), and autograft groups (64.36 ± 4.01%), these values were significantly higher (*p* < 0.01) than those in the hollow (43.88 ± 6.03%) and uASCs groups (45.81 ± 4.44%) (Fig. 7b). At the twelfth week after cell transplantation, the gastrocnemius on the injured side showed visible atrophy in the hollow and uASCs groups in comparison with the normal side. In contrast, the degree of gastrocnemius atrophy was less pronounced in the other three groups (Fig. 7a). The gastrocnemius wet weight ratio in intermittent dASCs (71.46 ± 2.78%) was significantly greater (*p* < 0.05) than those in the hollow (44.70 ± 3.51%), uASCs (49.00 ± 3.15%), and sustaining dASCs 7d groups (64.45 ± 2.91%) (Fig. 7c). Representative Masson’s trichrome staining results of the gastrocnemius are shown in Fig. 7a to evaluate recovery of denervated muscles. All groups showed hyperplasia of blue-stained collagen fibers distributed among muscle fibers stained in red. The mean cross-sectional area of gastrocnemius fibers in the intermittent dASCs group (1332.24 ± 60.17 μm^2^) was significantly greater (*p* < 0.01) than those in the hollow (568.04 ± 108.59 μm^2^), uASCs (807.80 ± 49.20 μm^2^), and sustaining dASCs 7d groups (1107.81 ± 82.86 μm^2^) (Fig. 7d).

### Electrophysiology

Representative CMAP oscillograms of each group at week 12 after cell transplantation are shown in Additional file 3 (Figure S3a). The amplitude ratio of CMAP in the intermittent dASCs group (73.52 ± 4.06%) was significantly lower (*p* < 0.01) than that in the autograft group (83.92 ± 7.40%), but was significantly higher (*p* < 0.05) than those in the hollow (47.45 ± 1.66%), uASCs (47.34 ± 2.49%), and sustaining dASCs 7d groups (64.67 ± 2.58%) (Additional file 3: Figure S3b). Although the latency ratio of CMAP in intermittent dASCs (1.37 ± 0.17) was not significantly different compared with those in the sustaining dASCs 7d (1.56 ± 0.10) and autograft groups (1.18 ± 0.06), the latter were significantly lower (*p* < 0.01) than those in the hollow (1.98 ± 0.23) and uASCs groups (1.98 ± 0.23) (Additional file 3: Figure S3c).

## Discussion

In this study, ASCs cultured in our laboratory showed good capacity for multipotent differentiation, including adipogenesis, osteogenesis, and chondrogenesis, as has been reported for other MSCs (Fig. 1a–d). ASCs expressed MSC surface markers and were negative for expression of hematopoietic stem cell markers (Fig. 1e). Previous studies have indicated biological advantages of ASCs with regard to immunomodulatory and self-renewal functions. Furthermore, the major protein secreted by ASCs, bFGF, was shown to be an essential factor for the induction of MSCs toward cells with a SC-like phenotype [36, 37]. It has been demonstrated previously that, although SCLCs induced by both BMSCs and ASCs could enhance peripheral nerve regeneration, ASCs had relatively lower invasiveness and higher yields than BMSCs, and ASCs could be rapidly amplified in vitro with lower immunogenicity [21]. In addition, ASCs were more resistant to apoptosis in vitro than BMSCs [38]. Taken together, these observations indicated that ASCs had extensive advantages in differentiation into SCLCs for repairing peripheral nerve injury compared with BMSCs.

Although it was reported previously that MSCs differentiate into SCLCs in a time-dependent manner, no studies have been performed using intermittent induction methods to differentiate MSCs into SCLCs, as for adipogenic differentiation [34, 35]. In the present study, we applied an intermittent induction method to differentiate ASCs into SCLCs in comparison with the traditional sustaining induction method. To examine the time dependency of traditional sustaining induction, we divided the traditional method into three time periods: sustaining dASCs 4d, 7d, and 10d (Fig. 1f).

The key difference between intermittent induction and traditional sustaining induction was the lack of bFGF and PDGF in the incomplete induction medium, which replaced complete induction medium on day 4 after sustaining induction and was then replaced by complete induction medium after 3 days. bFGF is a member of the FGF family, members of which are involved in a number of functions, including cell proliferation, migration, and differentiation, and are essential factors in tissue development, maintenance, and wound healing [39]. Some studies have shown that bFGF supplementation was associated with maintenance of pluripotency and inhibition of aging in MSCs [40]. Furthermore, bFGF is also a major inducer of vascularization in MSCs [41, 42], and upregulates the expression of PDGF receptors, which have a synergistic effect on the promotion of angiogenesis [43]. PDGF is a major mitogen for many types of mesenchymal cells, including fibroblasts and vascular smooth muscle cells [44]. In primary culture of immature SCs, PDGF promotes cell proliferation by activating Erk pathways, but inhibits the expression of pro-myelinating SC markers [45]. Moreover, the PDGF-PDGFR signaling system is involved in fibrosis and is believed to play a role in promoting the proliferation of cells with a fibroblast phenotype [46, 47]. Therefore, we believe that the sustained presence of bFGF and PDGF in the induction medium may preserve the multipotential differentiation of MSCs, such as adipocytes, fibroblast-like cells, endothelial-like cells, and even neuron-like cells, etc., thus reducing the induction efficiency of SCLCs.

Forskolin, an activator of adenylate cyclase extracted from the plant *Coleus forskholii*, is widely used in cell physiology to increase intracellular cyclic adenosine monophosphate (cAMP) levels and to promote cellular responses to neurotrophins [27, 48]. In some diseases that cause hypomyelination of the peripheral nervous system, the elevation of cAMP levels following treatment with forskolin is sufficient to restore myelination [49, 50]. This phenomenon highlights the importance of forskolin in the differentiation of myelinating SCs, and therefore the persistence of forskolin in the induction medium is beneficial to the maturation of SCLCs. HRG is a subtype of neuregulin, a genetic regulatory factor in the nervous system, and is also an important axon-derived signal that prevents the apoptosis of SC precursors, while regulating and controlling the formation of myelin and SCs [51, 52]. Therefore, the synergistic effect of HRG persisting throughout the whole induction process with forskolin can reduce apoptosis of SCLCs, which are successfully differentiated, and can induce SCLCs to differentiate into myelinating SCs to the maximum extent.

We also found that it is difficult to maintain the Schwann-like morphology of cells if they are always kept in incomplete induction medium. This result was consistent with the suggestion that bFGF is a crucial factor for maintaining Schwann-like morphology of cells [53]. Although the intermittent induction method achieved good results both in vitro and in vivo, the changes in cell signaling pathways induced by incomplete induction medium during differentiation remain unclear. We hypothesize that ASCs/SCLCs induce a transient memory of cellular differentiation during intermittent induction by incomplete induction medium, promoting ASC/SCLC differentiation into cellular phenotypes of transient memory more efficiently and stably on restimulation in complete induction medium.

Immunofluorescence staining showed that the rates of S100-positive cells increased in all groups with the length of induction period. Although the rate of S100-positive cells was higher in the intermittent dASCs group compared with the sustaining dASCs 4d and 7d groups, there was no significant difference between intermittent dASCs and sustaining dASCs 10d groups (Fig. 2a, b). However, the lack of difference between them was not absolute, because many of the sustaining dASCs that failed to differentiate into SCLCs underwent apoptosis, the number of surviving cells in sustaining dASCs groups decreased gradually with induction time, and then the rate of S100-positive cells increased indirectly (Fig. 2b, d). In fact, the success rate of differentiation in the sustaining dASCs 10d group was not only poorer than that of intermittent dASCs, it was even poorer than that of the sustaining dASCs 7d group. The number of NGFR p75-positive cells was not significantly different among all groups, even between uASCs and SCs (Fig. 2a, c). The high-affinity neurotrophic receptors, TrkA and TrkB, and the low-affinity neurotrophic receptor, NGFR p75, are major receptors of NGF [54, 55]. However, a recent study indicated that NGFR p75 and TrkA/TrkB exist not only in neurons and glial cells, but are also expressed in many other cell types, including MSCs [56]. As a member of the tumor necrosis factor receptor (TNFR) superfamily, it is generally accepted that NGFR p75 initiates apoptosis following cell injury [57]. This might explain the observed lack of difference in the rate of NGFR p75-positive cells between groups in the present study.

The qRT-PCR results showed that the level of NGFR p75 mRNA expression increased with the induction period and the maximum was reached on the seventh day of sustaining induction, with a subsequent decrease, which may have been related to negative feedback regulation after activation of the receptor. The level of NGFR p75 mRNA expression in the intermittent dASCs was not significantly different from those in sustaining dASCs 7d and SCs, which were higher than those in the other three groups. This may have been because cells in the intermittent dASCs group were in good condition, most NGFR p75 receptors had not been activated, and the cell apoptosis program had not been activated, leading to upregulation of NGFR p75 mRNA expression by negative feedback regulation (Fig. 3a). P0, the most important component of peripheral nerve myelin, is considered to be a crucial marker of matured SCs, which are myelinating Schwann cells [58]. In contrast, GFAP is a marker of non-myelinating SCs [16]. Intermittent dASCs showed a greater increase in P0 mRNA expression compared with sustaining dASCs 4d and 10d, but there was no significant difference compared with the sustaining dASCs 7d group (Fig. 3b). However, GFAP mRNA expression in intermittent dASCs was not different from those in all sustaining inducing groups (Fig. 3c). As expected, the above results indicated that the SCLCs of intermittent dASCs and sustaining dASCs 7d groups were closer to myelinating SCs at the transcriptional level than the other groups. This conclusion was also confirmed by in vivo experiments indicating that the density of myelinated nerve fibers, the mean diameter of myelinated nerve fibers, and the mean thickness of the myelin sheath in intermittent dASCs and sustaining dASCs 7d groups were superior to those in hollow and uASCs groups after cell transplantation (Fig. 6a–e).

qRT-PCR and ELISA results indicated that NGF expression at transcriptional and translational levels in the intermittent dASCs and sustaining dASCs 7d groups were significantly higher than those in uASCs and sustaining dASCs 4d and 10d groups (Additional file 2: Figure S2a and Fig. 3d). A previous study indicated that the presence of NGF in adipose tissue was crucial for the outgrowth of PC12 cell axons [59]. A similar phenomenon was also observed in the present study, in that DRG of the intermittent dASCs and sustaining dASCs 7d groups with higher NGF expression levels showed longer axon length than uASCs, and sustaining dASCs 4d and 10d groups in the Transwell® system (Fig. 4a, b). NGF also plays an important role in the development of nociceptive neurons of the DRG, as well as in the production and maintenance of neuropathic pain in a variety of experimental animal models of pain [60, 61]. At weeks 2 and 4 following cell transplantation, the stand/swing time ratios in the intermittent dASCs and sustaining dASCs 7d groups were significantly lower than those in the other three groups (Fig. 5a, c). This may have been because of the higher level of NGF secretion in these two groups at an early stage following cell transplantation, resulting in severe pain in the limb on the injured side, and thus the rats intentionally reduced the time that this limb touched the ground.

qRT-PCR and ELISA results also indicated equivalent BDNF expression both transcriptionally and translationally in the intermittent dASCs group and the sustaining dASCs 7d group, which were significantly higher than those in the uASCs group, other sustaining induction groups, and SCs (Additional file 2: Figure S2b and Fig. 3e). The BDNF/TrkB pathway is important for the formation of actin waves, which are indispensable for axon regeneration [62]. Simultaneously, BDNF increases cAMP levels during nerve regeneration, and upregulates the CREB-cjun-STAT3-Gap-43 pathway, which is associated with the intrinsic capacity of axonal growth. That is, BDNF delays degradation of distal nerves, while enhancing and maintaining the intrinsic capacity of axonal regeneration at a high level [63]. Finally, the above effects of BDNF were reflected in recovery of the SFI and neural electrophysiological evaluation in vivo. The SFI (Fig. 5a, b) and amplitude ratio of CMAP (Additional file 3: Figure S3a and b) in the intermittent dASCs group with a higher expression level of BDNF were similar to the autograft group, and significantly superior to the other groups.

CNTF from the chicken ciliary ganglion is a member of the interleukin-6 cytokine family [64, 65]. qRT-PCR and ELISA results showed that the intermittent dASCs and sustaining dASCs 7d groups had similar expression of CNTF at both transcriptional and protein secretion levels, which were significantly higher than those in the uASCs group and other sustaining induction groups (Additional file 2: Figure S2c and Fig. 3g). As an effective survival factor, CNTF can reduce tissue destruction during inflammation and protect damaged neurons from degeneration and necrosis [66]. This explains why the density of myelinated nerve fibers in intermittent dASCs and sustaining dASCs 7d groups were significantly greater than those in the hollow and uASCs groups at both the early stage (6 weeks after cell transplantation) and late stage (12 weeks after cell transplantation), but especially at the early stage (Fig. 6a–c). Moreover, CNTF has the ability to prevent scar tissue formation in the presence of other factors, such as BDNF [67]. This synergistic effect of CNTF with BDNF began to appear at 8 weeks after cell transplantation, resulting in the significantly higher stand/swing time ratio in the intermittent dASCs and sustaining dASCs 7d groups compared with the hollow and uASCs groups (Fig. 5c), indicating that the former two groups were less likely to generate neuropathic pain caused by hypertrophic scar formation during nerve regeneration. As a consequence, the rats in the intermittent dASCs and sustaining dASCs 7d groups did not intentionally reduce the time the limb on the injured side touched the ground, and the reduction of contact time was caused by haphalgesia.

GDNF was first discovered in the culture medium of glioma cell lines and is considered a member of the transforming growth factor-β (TGF-β) family [68]. Although GDNF is significantly more effective than BDNF in promoting axonal regeneration, a synergistic effect of BDNF and GDNF was observed in the treatment of tibial nerve injury in adult rats, which significantly increased the number of motor neurons in corresponding spinal segments [69]. At 6 weeks after cell transplantation, our study also indicated a guiding and synergistic effect through ultrasonic elasticity evaluation of the target organ, i.e., the gastrocnemius. Rats of the intermittent dASCs and sustaining dASCs 7d groups with higher expression levels of both GDNF and BDNF (Fig. 3e, f) had superior gastrocnemius elasticity than those in the hollow and uASCs groups (Fig. 7a, b). These observations indirectly demonstrated that the cells of intermittent dASCs and sustaining dASCs 7d groups were able to rapidly restore innervation of the target organ (gastrocnemius), thereby preventing muscle atrophy. Similarly, histological evaluation of the target organ (muscles) indirectly confirmed the guiding and synergistic effects of GDNF and BDNF. At 12 weeks after cell transplantation, both gastrocnemius wet weight ratio and cross-sectional area of gastrocnemius fibers of rats in the intermittent dASCs groups with higher expression of GDNF and BDNF (Fig. 3e, f) were superior to those in the other groups except the autograft group (Fig. 7a, c, d). GDNF can also relieve allodynia and hyperalgesia at the level of sensory neurons in the DRG [70]. In comparison with peptidergic neurons, which are NGF-dependent nociceptive neurons, the C fibers of non-peptidergic neurons in the DRG rely on the neurotrophin GDNF family as analgesic factors [61]. The mutual regulation between GDNF and NGF eventually resulted in an initial decrease in the stand/swing time ratio followed by an increase in the intermittent dASCs group (Fig. 5c).

## Conclusion

The results of the present study indicated that intermittent induction is a more efficient method of inducing the differentiation of ASCs into SCLCs in comparison with traditional methods of sustaining induction. In addition, SCLCs induced by this method were phenotypically close to mature myelinating SCs and were capable of secreting neurotrophins and promoting DRG axon regeneration in vitro. Furthermore, SCLCs induced by the intermittent induction method could repair sciatic nerve defects in rat models in vivo more effectively than traditional methods. Thus, intermittent induction provides a novel strategy for obtaining seed cells for use in nerve tissue engineering.

## Additional files

Additional file 1: **Figure S1.** Schematic diagram of the Transwell® system. DRGs were seeded in the lower chambers of the system, which included six 24-mm diameter, 0.4-μm pore polyester membrane inserts in a six-well plate. The uASCs, dASCs, and SCs were seeded in the upper inserts of the Transwell® system. (PDF 533 kb)

Additional file 2: **Figure S2.** Relative mRNA expression levels of neurotrophins in each group of cells. There were no significant differences in NGF (a), BDNF (b), and CNTF (c) mRNA expression levels between intermittent dASCs and sustaining dASCs 7d groups, but all were significantly higher (*p* < 0.01) compared with sustaining dASCs 4d and 10d groups. Data are expressed as means ± SEM. **p < 0.01, n.s. represents no significant difference, one-way ANOVA with Tukey’s post-test or Dunnett T3’s post-test. (PDF 1601 kb)

Additional file 3: **Figure S3.** Electrophysiological examination. (a) Representative CMAP oscillograms of each group at week 12 after cell transplantation. (b) The amplitude ratio of CMAP in the intermittent dASCs group (73.52 ± 4.06%) was significantly lower (*p* < 0.01) than that in the autograft group (83.92 ± 7.40%), but was significantly higher (*p* < 0.05) than those in the hollow (47.45 ± 1.66%), uASCs (47.34 ± 2.49%), and sustaining dASCs 7d groups (64.67 ± 2.58%). (c) Although the latency ratio of CMAP in intermittent dASCs (1.37 ± 0.17) was not significantly different compared with those in the sustaining dASCs 7d (1.56 ± 0.10) and autograft groups (1.18 ± 0.06), the latter were significantly lower (*p* < 0.01) than those in the hollow (1.98 ± 0.23) and uASCs groups (1.98 ± 0.23). Data are expressed as means ± SEM. *p < 0.05, **p < 0.01, n.s. represents no significant difference, one-way ANOVA with Tukey’s post-test or Dunnett T3’s post-test. (PDF 1201 kb)

## Abbreviations

α-MEM: α-Modified minimum essential medium
ASC: Adipose-derived stem cell
BDNF: Brain-derived neurotrophic factor
bFGF: Basic fibroblast growth factor
β-ME: β-Mercaptoethanol
BMSC: Bone marrow-derived stem cell
cAMP: Cyclic adenosine monophosphate
cDNA: Complementary DNA
CMAP: Compound muscle action potential
CNTF: Ciliary neurotrophic factor
DAPI: 4’,6-Diamidino-2-phenylindole
dASC: Differentiated adipose-derived stem cell
DMEM: Dulbecco’s modified Eagle’s medium
DMEM/F-12: Dulbecco’s modified Eagle’s medium/Ham’s F-12 50/50 Mix
DRG: Dorsal root ganglion
ELISA: Enzyme-linked immunosorbent assay
FBS: Fetal bovine serum
GAPDH: Glyceraldehyde 3-phosphate dehydrogenase
GDNF: Glial cell line-derived neurotrophic factor
GFAP: Glial fibrillary acidic protein
HRG: Recombinant human heregulin-β1
MSC: Mesenchymal stromal cell
NF200: Neurofilament protein 200
NGF: Nerve growth factor
NGFR p75: Nerve growth factor receptor p75
P0: Myelin protein zero
PBS: Phosphate-buffered saline
PDGF: Platelet-derived growth factor
PNI: Peripheral nerve injury
qRT-PCR: Quantitative reverse transcription polymerase chain reaction
RA: Retinoic acid
SCLC: Schwann cell-like cell
SC: Schwann cell
SD: Sprague–Dawley
SFI: Sciatic functional index
SWE: Shear wave elastography
TEM: Transmission electron microscopy
uASC: Undifferentiated adipose-derived stem cell
