# Supplementary material for: Differentiation of adipose-derived stem cells into Schwann cell-like cells through intermittent induction: potential advantage of cellular transient memory function
Source: Stem Cell Res Ther. 2018 May 11;9:133. doi: 10.1186/s13287-018-0884-3 (PMC5948899; doi:10.1186/s13287-018-0884-3)
Supplement: Supplementary file 1 — Figure S1. Schematic diagram of the Transwell® system. DRGs were seeded in the lower chambers of the system, which included six 24-mm diameter, 0.4-μm pore polyester membrane inserts in a six-well plate. The uASCs, dASCs, and SCs were seeded in the upper inserts of the Transwell® system. (PDF 533 kb) [file 13287_2018_884_MOESM1_ESM.pdf]

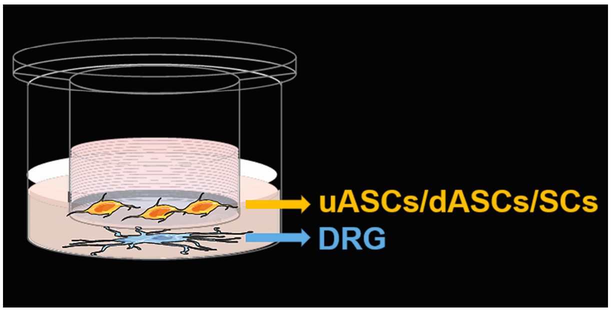

Additional file 1: Figure S1. Schematic diagram of the Transwell® system. DRGs were seeded in the lower chambers of the system, which included six 24-mm diameter, 0.4-µm pore polyester membrane inserts in a 6-well plate. The uASCs, dASCs and SCs were seeded in the upper inserts of the Transwell® system.
