# Supplementary material for: Differentiation of adipose-derived stem cells into Schwann cell-like cells through intermittent induction: potential advantage of cellular transient memory function
Source: Stem Cell Res Ther. 2018 May 11;9:133. doi: 10.1186/s13287-018-0884-3 (PMC5948899; doi:10.1186/s13287-018-0884-3)
Supplement: Supplementary file 2 — Figure S2. Relative mRNA expression levels of neurotrophins in each group of cells. There were no significant differences in NGF (a), BDNF (b), and CNTF (c) mRNA expression levels between intermittent dASCs and sustaining dASCs 7d groups, but all were significantly higher (p < 0.01) compared with sustaining dASCs 4d and 10d groups. Data are expressed as means ± SEM. **p < 0.01, n.s. represents no significant difference, one-way ANOVA with Tukey’s post-test or Dunnett T3’s post-test. (PDF 1601 kb) [file 13287_2018_884_MOESM2_ESM.pdf]

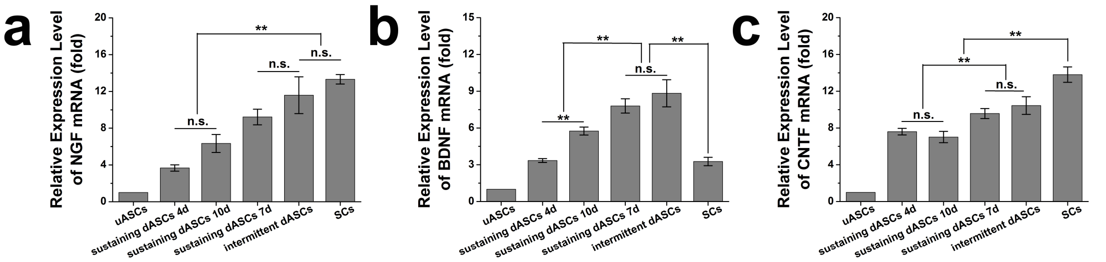

Additional file 2: Figure S2. Relative mRNA expression levels of neurotrophins in each group of cells. There were no significant differences in NGF (**a**), BDNF (**b**) and CNTF (**c**) mRNA expression levels between intermittent dASCs and sustaining dASCs 7d groups, but all were significantly higher ($p < 0.01$) compared with sustaining dASCs 4d and 10d groups. Data are expressed as means ± SEM. **$p < 0.01$, n.s. represents no significant difference, one-way ANOVA with Tukey's post-test or Dunnett T3's post-test.
