# Supplementary material for: Differentiation of adipose-derived stem cells into Schwann cell-like cells through intermittent induction: potential advantage of cellular transient memory function
Source: Stem Cell Res Ther. 2018 May 11;9:133. doi: 10.1186/s13287-018-0884-3 (PMC5948899; doi:10.1186/s13287-018-0884-3)
Supplement: Supplementary file 3 — Figure S3. Electrophysiological examination. (a) Representative CMAP oscillograms of each group at week 12 after cell transplantation. (b) The amplitude ratio of CMAP in the intermittent dASCs group (73.52 ± 4.06%) was significantly lower (p < 0.01) than that in the autograft group (83.92 ± 7.40%), but was significantly higher (p < 0.05) than those in the hollow (47.45 ± 1.66%), uASCs (47.34 ± 2.49%), and sustaining dASCs 7d groups (64.67 ± 2.58%). (c) Although the latency ratio of CMAP in intermittent dASCs (1.37 ± 0.17) was not significantly different compared with those in the sustaining dASCs 7d (1.56 ± 0.10) and autograft groups (1.18 ± 0.06), the latter were significantly lower (p < 0.01) than those in the hollow (1.98 ± 0.23) and uASCs groups (1.98 ± 0.23). Data are expressed as means ± SEM. *p < 0.05, **p < 0.01, n.s. represents no significant difference, one-way ANOVA with Tukey’s post-test or Dunnett T3’s post-test. (PDF 1201 kb) [file 13287_2018_884_MOESM3_ESM.pdf]

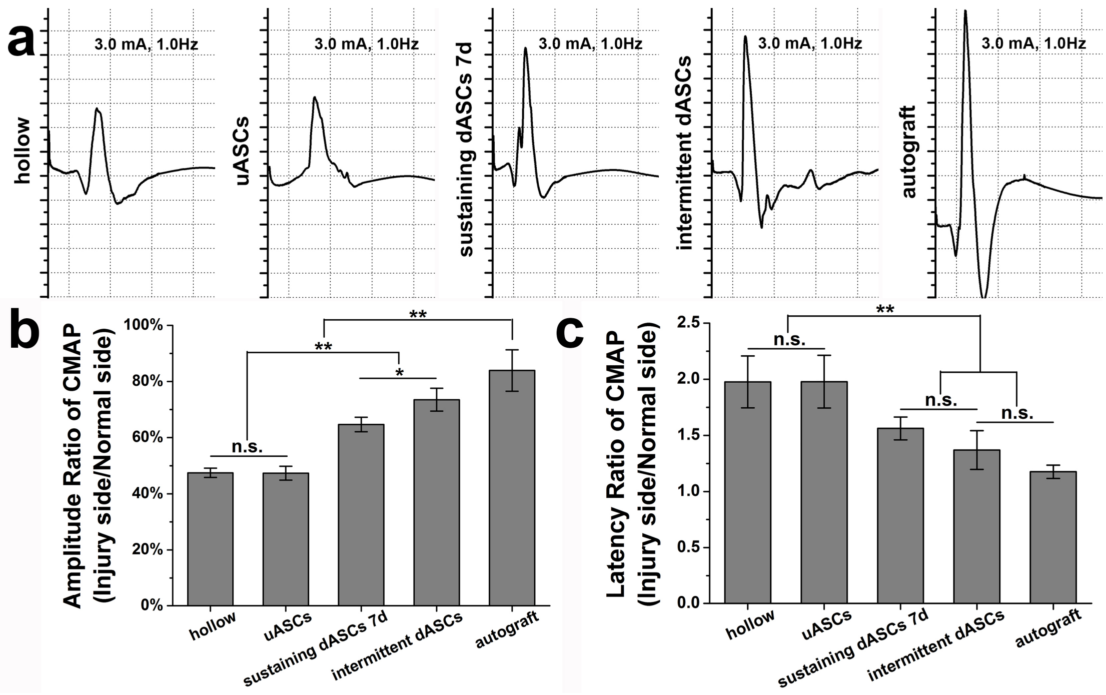

Additional file 3: Figure S3. Electrophysiological examination. **a** Representative CMAP oscillograms of each group at week 12 after cell transplantation. **b** The amplitude ratio of CMAP in the intermittent dASCs group (73.52 ± 4.06%) was significantly lower ($p < 0.01$) than that in the autograft group (83.92 ± 7.40%), but was significantly higher ($p < 0.05$) than those in the hollow (47.45 ± 1.66%), uASCs (47.34 ± 2.49%), and sustaining dASCs 7d groups (64.67 ± 2.58%). **c** Although the latency ratio of CMAP in intermittent dASCs (1.37 ± 0.17) was not significantly different compared with those in the sustaining dASCs 7d (1.56 ± 0.10) and autograft groups (1.18 ± 0.06), the latter were significantly lower ($p < 0.01$) than those in the hollow (1.98 ± 0.23) and uASCs groups (1.98 ± 0.23). Data are expressed as means ± SEM. *$p < 0.05$, **$p < 0.01$, n.s. represents no significant difference, one-way ANOVA with Tukey's post-test or Dunnett T3's post-test.
